# Genome Sequence of the Aerobic Arsenate-Reducing Bacterium *Pantoea* sp. Strain IMH

**DOI:** 10.1128/genomeA.00267-14

**Published:** 2014-04-10

**Authors:** Haixia Tian, Chuanyong Jing

**Affiliations:** State Key Laboratory of Environmental Chemistry and Ecotoxicology, Research Center for Eco-Environmental Sciences, Chinese Academy of Sciences, Beijing, China

## Abstract

We here report the draft assembly for the genome of *Pantoea* sp. strain IMH, isolated from arsenic-contaminated soil in Inner Mongolia, China, with the ability to aerobically reduce arsenate to arsenite. The genome sequence will allow for the characterization of the molecular mechanisms of arsenate reduction.

## GENOME ANNOUNCEMENT

The genus *Pantoea* comprises a number of plant pathogens (1). Generally, the pathogenicity of the genus *Pantoea* has been the major focus. In 2013, for the first time, the *Pantoea* genus was reported to reduce arsenate [As(V)] to more toxic arsenite [As(III)] and play an important role in the arsenic biogeochemical cycle (2). *Pantoea* sp. strain IMH was isolated from arsenic-contaminated soil in Inner Mongolia, China. Strain IMH was highly resistant to As(V) (with an MIC of 150 mM) and reduced over 90% As(V) in 36 h. Previous studies indicate that aerobic As(V) reduction is mostly regulated via chromosomal or plasmid-carried *ars* operons. The five-gene *arsRDABC* and three-gene *arsRBC* are the two most common types of *ars* operons (3). However, the genomic information for strain IMH is not fully understood (2). Therefore, we chose to sequence the genome of strain IMH to explore how arsenate reduction occurs.

The genome of strain IMH was sequenced using the Illumina HiSeq 2000 sequencing platform at the Beijing Genomics Institute (BGI) (Shenzhen, China). Two libraries containing 500 bp and 6,000 bp were constructed. Sequencing was performed with the paired-end strategy of (90, 90)-bp reads to produce 754 Mb of filtered sequences. The genome of strain IMH consists of a single chromosome ~4.09 Mb in size, with a G+C content of 54.74%. There are two scaffolds and eight contigs.

Genes were predicted from the assembled result using Glimmer 3.02 (4). Genome annotation was accomplished by analysis of protein sequences. The resulting translations were aligned with databases, including KEGG 59 (5), GO 1.419 (6), and Swiss-Prot 201206 (7). The genome contains 3,875 candidate protein-encoding genes (with a total length of 3,500,973 bp), giving a coding intensity of 85.57%. Eighty-one tRNAs and 23 rRNA operons were identified.

In particular, we analyzed the genes possibly responsible for As(V) reduction. Four pertinent genes (*arsH*, *arsC*, *arsB*, and *arsR*) involved in regulating As(V) reduction and resistance were identified in the genome. Comparison of the *arsC* gene of strain IMH to those of *Pantoea ananatis* LMG 20103 (GenBank accession no. CP001875) and *Pantoea ananatis* AJ13355 (accession no. AP012032) showed that the similarities were 88% and 98%, respectively. Among these *Pantoea* strains, only strain IMH has the *arsH* gene. In addition, numerous genes responsible for metal-ion binding and transport were also identified.

### Nucleotide sequence accession numbers.

This whole-genome shotgun project has been deposited at GenBank under the accession number JFGT00000000. The version described in this paper is version JFGT01000000.
